# Reflective interventions for cybersecurity: insights from a sociotechnical framework application and assessment

**DOI:** 10.1007/s10111-025-00833-6

**Published:** 2025-09-29

**Authors:** Neeshe Khan, Sarah Sharples, Robert J. Houghton

**Affiliations:** 1 University of Nottingham, Nottingham, UK; 2 University of Manchester, Manchester, UK

**Keywords:** Cyber security, Human Factors, Unintentional insider threat, theory of planned behaviour, framework validation, organisations

## Abstract

Managing unintentional insider threat (UIT)—inadvertent mistakes and errors that cause cyber breaches—remains a serious challenge to organisations and businesses. In previous work (Khan et al. in Cognit Technol Work 24:1–29, 2021) we developed a sociotechnical framework for UIT consisting of 45 elements arranged in six categories called ‘pillars’ (for e.g. technical cyber defences, user vulnerabilities, processes, knowledge sharing etc.). In the present paper we report the use of a web-based assessment tool that embodied this model and conducted a mixed methods study to examine its effectiveness as a tool for reflecting on these challenges and the consideration of future organisational responses. Senior leaders were invited to engage with the web-based assessment tool (hosted via a website) for a three-hour session to explore the application of the previously developed sociotechnical framework to identify where participants believed their organisation lay in terms of maturity. Attitudes were assessed through semantic scales and semi-structured interviews pre- and post-session that used the lens of Ajzen’s Theory of Planned Behaviour to explore attitudes, subjective norms and perceived control around the cybersecurity issues identified. It was found that organisations view informal peer structures as beneficial and invest in individual development if it is relevant to the job function already being performed by individuals. Organisations also showed aspirations to continuously improve the state of their technical and sociotechnical defences through investing in people and better technologies. Potential areas for improvement of the assessment tool’s inputs were also identified. We conclude that the web-based tool that was developed from the framework is an effective intervention to change planned behaviour for safeguarding against UIT. More broadly, the work demonstrates how an empirically derived framework for understanding human behaviour can be extended as a tool for reflection and determining future actions to improve organisational safety and security measures.

**Supplementary Information:**

The online version contains supplementary material available at 10.1007/s10111-025-00833-6.

## Introduction

The unwitting facilitation and unintended actions by individuals that result in cyber breaches are classified as a subset of ‘Insider Threat’ known as unintentional or accidental insider threat (UIT). Between 2020 and 2022, the costs associated to insider threat related breaches has risen by 44%, with each incident estimated to cost $15.38 m (USD) and taking an average of 81 days to contain the incident (Ponemon Institute 2022a, 2022b). Previous work to tackle UIT can be classified as emphasising two main forms of prevention namely, technical defences and sociotechnical defences. Technical defences involve algorithm-based solutions and may incorporate elements of automation, machine learning and artificial intelligence to block or challenge unsafe behaviour (e.g., Morel 2011). In contrast, sociotechnical defences emphasise a systems approach concerned with people, organisations, technology and their interactions. Some sociotechnical defences have extended the use of human factors approaches associated with safety critical work to create sociotechnical cyber defence. For example, Liginlal (2009) proposes an approach based around the GEMS model (Reason 1990).

In this article we further previous work by Khan et al. (2021) that proposes a sociotechnical framework to counteract UIT. A web-based tool (hosted via a website) was developed to assess organisational defences’ readiness levels through evaluating 45 inputs associated to six categories referred to as pillars. The six pillars were namely, technical cyber defences; user vulnerabilities; processes; workload and resource allocation; knowledge sharing and empowerment; and fluctuating vulnerabilities. By applying the Theory of Planned Behaviour (Ajzen 1991), behaviour changes amongst our participants were measured to investigate whether the framework can enhance existing sociotechnical defences, especially those defences that safeguard against UIT. In order to explore the effectiveness of the framework, we begin by briefly discussing relevant literature that informs the design of technical and sociotechnical solutions to safeguard against UIT and explain the framework previously developed from authors’ previous work.

## Background

Insider threat (IT) can be understood as a threat to systems and infrastructure due to the action or inaction of individuals within organisations or ‘insiders’. IT becomes a critical vulnerability due to the insider’s access privileges and working knowledge of systems. Severity of this type of threat is further affected by insider’s skillset and motivations. Predd et al. (2008) discuss IT as being comprised of two categories: intentional and unintentional (UIT), both of which can be posed by an individual or a group. UIT is of interest to this work at least in part because of their familial relationship with elements of safety science; deliberate malevolent acts lie outside the scope of this work. UIT is accidental in its nature and thus arises from well-meaning or unintentional actions or inactions by insiders that can cause harm to assets, operations or the larger system.

Technical cyber defences have enjoyed popularity for their perceived ease in circumventing IT, which arguably have limited effect for tackling UIT due to its unintended nature. As part of the larger Defensive Cyberspace Operations (DCOs) approaches, which stem from traditional security thought, passive and active defences are implemented by organisations (Ani et al. 2018; Goethals and Hunt 2019). Technical defences can also encompass algorithmic solutions that consider insiders’ psychological and behavioural traits to identify and predict threats (Legg et al. 2015; Greitzer and Hohimer 2011).

Safeguards that incorporate factors at an individual and organisational level in addition to aforementioned technical elements are known as sociotechnical defences. Sociotechnical defences are designed to tackle IT in complex environments and take human factors into account. For instance, ‘10 steps to cybersecurity’ (NCSC 2012) proposes technical defences such as network security, malware prevention software, secure configuration and management of user privileges. Individual level defences include monitoring user activity and devices. Organisational defences within this guide comprise of educating individuals and training programmes based on organisational security policies, up-skilling individuals through formal qualifications, effective communications and instilling a no-blame culture.

MERIT is another notable sociotechnical defence framework by CERT (Keeney et al. 2005; Cappelli et al. 2007, 2008). MERIT proposes the use of passive cyber defences as well as human based defences for instance, evaluating motivations (through individual psychological and behaviour profiling), monitoring skillsets and assessing opportunities afforded to insiders. Psychological profiling through the use of personality traits has been included in several other sociotechnical frameworks to detect and safeguard against UIT (Hadlington 2018; Greitzer et al. 2018). Organisational factors within the MERIT framework include training programmes, robust security policies, interventions for undesirable behaviour and management of expectations. In subsequent work, CERT (2013) connects UIT to human fallibility and performance limitations which arise due to a number of factors. These factors include time pressures to deliver tasks, lack of knowledge, level of difficulty associated to a task and cognitive load induced by tasks being performed by individuals. Furthering CERT’s research, Nurse et al. (2014) propose a sociotechnical framework to tackle IT. Within Nurse et al.’s framework UIT is associated to factors (such as time pressures), but these factors are believed to emanate from task objectives.

Liginlal et al. (2009) created a sociotechnical framework, known as the error management programme, to tackle IT through extending the application of Generic Error-Modelling System (GEMS). This GEMS model by Reason (1990) classifies human errors through examining cognitive loads associated to tasks. Human errors are considered in isolation from environmental or other context related factors. Unsafe actions and decisions are believed to originate from unintentional or intentional actions which subsequently result in undesirable outcomes. Undesirable outcomes or errors are classified into four categories: slips (from attention failures), lapses (in memory), mistakes (rule or knowledge based) and violations (from unsafe routines or due to novel application in exceptional circumstances). Framed by this understanding error management programme examines root causes that lead to errors, proposes creation of defence strategies that avoid, intercept and correct errors and recommends evaluating processes periodically for effectiveness. Liginlal et al. (2009) framework also recommends training programmes, effective design which includes displays, monitoring and alarms, timely investigation of errors, a no-blame organisational culture, careful consideration prior to implementing new systems, effective processes, and monitoring work related fatigue.

### Approach

Authors’ previous work involved the application of Critical Decision Method (Klein et al. 1989) to the accounts of individuals who had inadvertently participated in data breaches (Khan et al. 2021). Analysis of this data revealed six main pillars which potentially influence UIT: Technical, User Vulnerabilities, Processes, Workload and Resources, Knowledge, and Fluctuating Vulnerabilities. Additionally, a set of linked concepts for security improvement were identified including, homing in expert staff when devising processes, monitoring factors linked to time pressures, known channels of knowledge attainment and sharing and, monitoring fluctuating vulnerabilities linked to UIT (Table 7; Khan et al. 2021).

The present work is primarily focused on whether this empirically derived framework could be extended in a manner that allowed it to be used as a focus for reflection on cybersecurity issues at an organisational level and whether it could guide organisational decision making. We take inspiration from previous theory-to-practice ventures including the use of Reason’s Swiss Cheese Model of accident causation taking form as an organisational decision-making tool (e.g., Wiegmann and Shappell 2003).

In order to understand how the framework informed perceptions of organisational status around unintentional cybersecurity threat, we adopted Ajzen’s Theory of Planned Behaviour as an analytic lens. The theory proposes three independent determinants to predict certain behaviours namely, attitude towards the behaviour, subjective norms and perceived behaviour control to achieve desired outcomes. The Theory of Planned Behaviour has been applied in several domains including environmental sciences, occupational health domain, management (Bosnjak et al. 2020) leisure choice (Ajzen and Driver 1992) and continuing higher education (Ingram et al. 2000). We measured these three determinants within our participants prior to and post interacting with our web-based assessment tool, which evaluated factors that influence unintentional insider threat (UIT) within organisations. The three determinants that indicate changes in behaviour were selected to investigate whether the tool can serve as an intervention against future UIT for organisations of various sizes.

We investigated attitudes, subjective norms and perceived behaviour control amongst our participants through the application of metacognition scaffolding technique (Jumaat and Tasir 2014), seven-point semantic scales, feeling thermometers and open-ended questions to inform semi-structured interviews (Rydell and Macconnell, 2006). Participants were also asked to reflect and verbally share any new learnings and assess their progress post their interaction with the tool. This resulted in rich data garnered from in-depth conversations.

### Website development

**Fig. 1:**
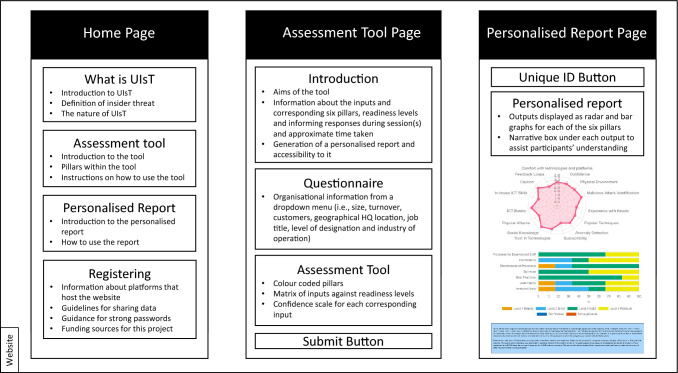
Website design containing three core pages

The web-based assessment tool was hosted via a website which contained three core pages (shown in Fig. 1); 1. Home Page which provided definitions for ‘Insider Threat’ and ‘Unintentional Insider threat’, information about the ‘Assessment tool’, information about the ‘Personalised Report’, and information about ‘registering with the website’; 2. Assessment Tool Page which provided an introduction to the tool, a brief questionnaire about the organisation (e.g. “Size of the organisation”, “Please describe the nature of your customers”, “Please indicate your current function” and “Please indicate the management level of your current position”) providing multiple options for response. This page also included six colour coded pillars which reflected the readiness levels on a four-point scale against each of the factors that were found to influence UIT (Khan et al. 2021), a three-point semantic confidence scale for each of the inputs to capture participants’ confidence levels when selecting their organisational readiness levels, and a ‘submit’ button for when the assessment tool was completed; 3. Personalised ‘Report Page’ which displayed the strength of participants’ organisational defences against UIT based on their choices through visuals such as radar and bar graphs for each of the pillars and a narrative box to assist participants in interpreting the visualisations.

## Method

### Participants

Thirteen participants were recruited from six organisations with the help of multiple National Cyber Security Centre’s (NCSC) ‘Industry 100’ organisational partners and through the first author’s professional network. All participants from the same organisation were present concurrently during their respective session(s). As shown in Table 1, six participants held a Chief Executive Officer or equivalent designation, six participants were at a Director, Head or equivalent designation, whilst one participant held a Manager or equivalent title. Two participating organisations were small or medium-sized enterprise (SMEs), two were large and two were non-profit organisations. Four organisations indicated the primarily nature of their customers as being ‘business-to-business’ (B2B) whilst two indicated ‘business-to-consumers’ (B2C) where products or services are taken directly to end users. Organisational headquarters (HQ) were either located in the United Kingdom or Americas region.

**Table 1:** Demographic information of interview participants

Participant ref	Company ref	Role equivalate	Size of organisation	Nature of customers	Regional headquarter location
AL	1	Chief Executive Officer	Small or medium-sized enterprise (SME)	B2B	United Kingdom
GL	1	Head	Small or medium-sized enterprise (SME)	B2B	United Kingdom
BP	2	Chief Executive Officer	Small or medium-sized enterprise (SME)	B2B	United Kingdom
SN	2	Head	Small or medium-sized enterprise (SME)	B2B	United Kingdom
MM	3	Chief Executive Officer	Large enterprise	B2B	Americas
HR	3	Chief Operating Officer	Large enterprise	B2B	Americas
RR	4	Director (Global)	Large enterprise	B2B	Americas
KL	4	Head	Large enterprise	B2B	Americas
ST	5	Director	Non-profit organisation	B2C	United Kingdom
MK	5	Chief Executive Officer	Non-profit organisation	B2C	United Kingdom
DS	5	Manager	Non-profit organisation	B2C	United Kingdom
GH	6	Director	Non-profit organisation	B2C	United Kingdom
KK	6	Head	Non-profit organisation	B2C	United Kingdom

*B2B, Business-to-business; B2C, Business-to-consumer

### Data collection

Nine sessions took place between January and March 2022; the longest session was 3 h 03 min, with the shortest lasting 1 h 55 min. Sessions cumulatively generated approximately 14 h and 30 min of data.

Data was collected in four stages. Initially participants were required to complete a questionnaire which contained free text fields and a seven-point semantic scale. Following this initial stage, data was captured during the session through audio and video recording as well as through the website which contained information about participants’ organisation, recorded their selections for readiness levels against UIT and confidence levels on a three-point semantic scale. Once participants had interacted with the website which contained 45 inputs across six pillar and studied their organisation’s personalised report, participants were asked to fill out another questionnaire, similar to the one they had filled out prior to the session. Lastly, data was collected through semi-structured interviews conducted at the end of session(s).

### Session design

Prior to the session participants filled out a short questionnaire sheet containing sixteen questions. Three questions contained a free text field for participants’ responses. For the remainder of the thirteen questions, participants were asked to select their agreement levels pertaining to statements shown in the question on a seven-point semantic scale ranging from “Strongly Disagree” to “Strongly Agree”. Full set of questions within the questionnaire sheet that measured participants’ attitudes (Q1–Q6), subjective norms (Q7–Q11) and perceived behaviour control (Q12–Q16) as outlined in the Theory of Planned Behaviour can be found in Online Appendix A.

During the session participants were reminded of the nature, purpose and duration of this study, their rights, and offered a chance to ask questions before proceeding. Participants were then informed of the structure of the session, including ten-minute breaks on the hour if needed.

The session began with the senior stakeholder from each organisation sharing their screen. Accompanying participants and the first author conducting the session (referred to as ‘IV’) confirmed they could see and read the content being displayed. The senior stakeholder was directed to a website which contained three core pages; Home Page, Assessment Tool page, and Personalised Report page. Participants were given time to read over the information presented on the Home Page before progressing on to the Assessment Tool. On average, it took participants 55 min to complete the assessment tool (shortest time: 30 min; longest time: 1 h 15 min). All attending participants from the same organisation were requested to agree on the readiness level selected for their organisation for each of the inputs, with the senior stakeholder having the final decision. Due to the nature of the business for some of the participating organisations, all participants were informed that they did not need to share any confidential information or reasoning for their selections in front of the interviewer if they did not feel comfortable. However, participants appeared to be comfortable and engaged in ‘thinking out loud’ technique as they progressed through their selections.

After participants submitted their choices, they were given time to independently look at and interpret the personalised report generated for their respective organisations. Once the personalised report had been interpreted, participants were requested to fill out and return another short questionnaire sheet containing sixteen questions, similar to the one that was filled out prior to the session, shown in Online Appendix B (attitudes: Q1–Q6; subjective norms: Q7–Q11; perceived behaviour control: Q12–16). Once the questionnaire sheets were returned, participants were asked nine open-ended questions as part of a semi-structured group interview, which can be found in Online Appendix C (metacognition scaffolding technique: Q1–2; Q4–9; perceived behaviour control: Q3). On average, semi-structured interviews lasted 54 min (shortest time: 34 min; longest time: 1 h 15 min).

**Fig. 2:**
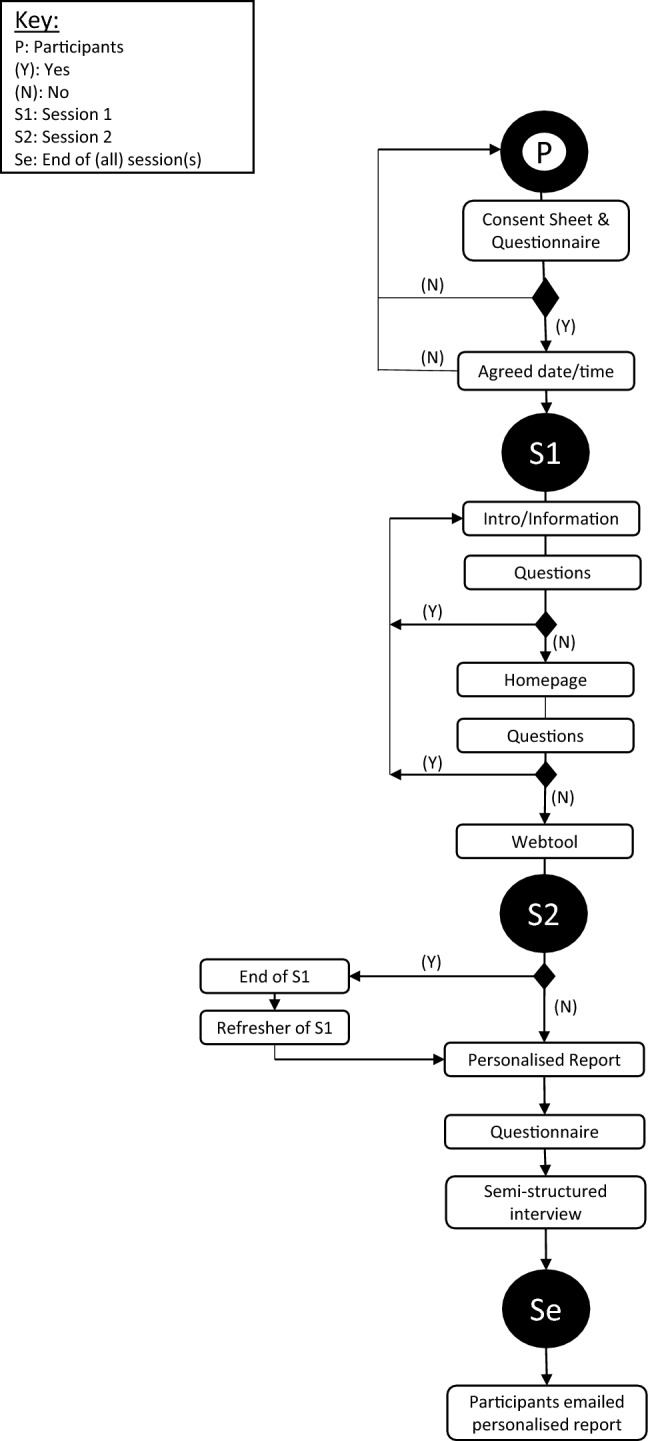
Flow diagram of activities within session(s)

Post the session, participants received an email of gratitude for their time which contained a PDF copy of their personalised report and authors’ contact details should they have any queries in the future (Fig. 2).

### Analysis

Quantitative data from questionnaires circulated to participants prior to and during their session(s) was compiled in a table format and colour coded by themes to respectively represent the question’s category (i.e. attitudes, subjective norms and, control). Any changes to the type of words used to answer free text fields were observed. Responses to the semantic scales were averaged, the range calculated (i.e. the lowest and highest selected points within the semantic scale prior and during sessions) and, any outliers were noted. The range in responses was noted in order to provide context for overall change in behaviour and attitudes during session(s). This is shown in Table 2, along with author notes to assist in interpretation of findings. This analysis and subsequent conclusion was done by the first author and independently validated by the last author of this article.

**Table 2:** Average and range of responses to semantic scale questions

Theme	Q. No	Pre-session avg. response	Range of rating	Post-session avg. response	Range of rating	Author notes
Attitude	Q. 3 Training and technical cyber defences are the main way to protect against UIT	3.9	5	3.6	6	This question shows an overall downward shift on the semantic scale towards ‘Strongly Disagree’. 6 participants changed their initial ratings. An outlier is noted [GH:6] with a four point upward shift on the scale
			(Lower: 2 Upper: 7)		(Lower: 1 Upper: 7)	
Attitude	Q. 4 Cybersecurity vulnerabilities are static over time	2.9	5	3.2	5	This question shows an overall upward shift on the semantic scale towards ‘Strongly Agree’. Four participants changed their initial ratings. An outlier is noted [KK:6] with a five point upward shift on the scale
			(Lower: 1 Upper: 6)		(Lower: 1 Upper: 6)	
Attitude	Q. 5 UIT is a direct consequence of rule breaking behaviour	4.6	4	4.2	4	This question shows an overall downward shift of towards ‘Strongly Disagree’. Four participants changed their initial ratings
			(Lower: 2 Upper: 6)		(Lower: 2 Upper: 6)	
Attitude	Q. 6 Cybersecurity is mainly concerned with computer-based interactions	3.7	5	3.5	6	This question shows an overall downward shift towards ‘Strongly Disagree’. Four participants changed their initial ratings. An outlier is noted [KL:4] with a four point downward shift on the scale
			(Lower: 1 Upper: 6)		(Lower: 1 Upper: 7)	
Subjective norms	Q.7 Organisational knowledge sharing will increase in the future	5.0	5	5.7	4	This question shows an overall upward shift of towards ‘Strongly Agree’. One participant went down by a point
			(Lower: 2 Upper: 7)		(Lower: 3 Upper: 7)	
Subjective norms	Q.8 Organisational stakeholder interest in learning about insights regarding cyber defences	6.5	2	5.2	3	This question shows a downward shift towards ‘Strongly Disagree’. While a majority of the participants strongly agreed with this question initially, they did not think that there’d be wider interest in their organisations on this topic or in the findings. While everyone is believed to be responsible, people are not believed to be actively interested
			(Lower: 5 Upper: 7)		(Lower: 3 Upper: 6)	
Subjective norms	Q.9 Exploration of insights gathered to improve and inform defences	5.3	5	5.8	3	This question shows an upward shift towards ‘Strongly Agree’. The subjective norms in place led participants to believe that others take action and they themselves are able to do so
			(Lower: 2 Upper: 7)		(Lower: 4 Upper: 7)	
Subjective norms	Q.10 Support of cyber initiatives from senior stakeholders	6.2	3	6.1	3	Overall participants believed that they were seen to be responsible for making their organisations cyber resilient. Eight participants ‘Strongly Agreed’ with getting support from senior stakeholders whereby only two positively changed their ratings after the session. Five participants didn’t indicate that they would receive strong support from senior stakeholders with an overall downward shift towards ‘Strongly Disagree’
			(Lower: 4 Upper: 7)		(Lower: 4 Upper: 7)	
Subjective norms	Q.11 Consideration of workload (WL), procedures and resources when creating cybersecurity practices	5.2	6	5.8	4	Responses showed an upward shift towards ‘Strongly Agree’. Participants indicated subjective norms of being able to understand and consider WL, procedures and resources. Participants’ subjective norms appeared to be supportive of developing this understanding when creating cybersecurity practices
			(Lower: 1 Upper: 7)		(Lower: 3 Upper: 7)	
Control	Q.13 Evaluation of existing procedures and practices to enhance cybersecurity	5.3	6	6.5	1	This question shows an upward shift of towards ‘Strongly Agree’. Participants indicated control over the design of procedures and processes (in belief and in practice). Participants also indicated a positive correlation between procedures/practices and cybersecurity with all ratings towards ‘Strongly Agree’
			(Lower: 1 Upper: 7)		(Lower: 6 Upper: 7)	
Control	Q.14 Ability to form groups to enhance information and experiences	6.0	3	5.3	6	This question shows a downward shift towards ‘Strongly Disagree’. While participants showed control over being able to start new group activities, this control belief didn’t permeate into forming a group in real world setting
			(Lower: 4 Upper: 7)		(Lower: 1 Upper: 7)	
Control	Q.15 Ability to gain interest from senior stakeholders in cyber defence discoveries	6.5	2	5.6	4	This question shows a downward shift towards ‘Strongly Disagree’. While participants believed that senior stakeholders would be interested in their experiences and information they provided, participants did not feel that they could control what the senior board would be interested in and unable to influence board’s strategies
			(Lower: 5 Upper: 7)		(Lower: 3 Upper: 7)	
Control	Q.16 Technological defences to safeguard against UIT	3.4	4	4	5	Responses indicated an upward shift towards ‘Strongly Agree’. One outlier is noted [SN:2] with a 6 point upward shift. Participants expressed control through restriction of users’ access in IT systems in order to avoid UIT
			(Lower: 1 Upper: 5)		(Lower: 2 Upper: 7)	

All questions utilising a seven-point semantic scale from ‘strongly disagree’ to ‘strongly agree’ are presented below alongside their associated themes. Responses were averaged and the range was determined to capture overall changes in attitudes and behaviour prior and during session(s). Author’s notes are also shown below to provide context to the findings

Session recordings were transcribed verbatim by the first author. All transcripts were anonymised and parts redacted where appropriate to safeguard participants’ anonymity. Clean transcripts were uploaded to QSR-NVivo software for coding qualitative data. Template analysis (King 2012) was used whereby a template containing broad themes i.e. parent nodes along with nested child nodes were utilised to code data (top-down approach). With the application of grounded theory Glaser and Strauss (1967) to the transcripts, the initial template was updated with new parent and child nodes as they emerged (bottom-up approach). Template analysis provides researchers flexibility in procedures for data gathering and analysis to match their own objectives. This approach also affords time efficiency compared to other approaches (e.g. interpretive phenomenological analysis or IPA, Smith and Shinebourne 2012), is not infused with a particular methodological or theoretical position and allows researchers flexibility within the coding structure.

## Results

**Fig. 3:**
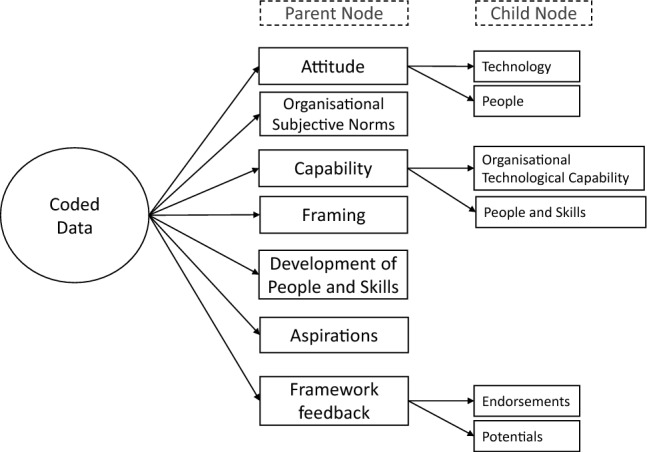
Thematic distribution of data

Seven themes that emerged from data, shown in Fig. 3, were indicative of attitudes, subjective norms and perceived behaviour control (Ajzen 1991) and contained elements of reflective learning amongst the participants post their interaction with the website. Three parent nodes derived from the Theory of Planned Behaviour were namely: ‘Attitudes’ which reflected participants’ beliefs regarding technology and people; ‘Organisational subjective norms’ which was indicative of participants’ normative beliefs and their motivations to comply in their respective organisations; and ‘Capability’ which was indicative of perceived control reflected through participants’ beliefs regarding opportunities, resources and self-efficacy on an individual and an organisational level. The remaining parent nodes, i.e. Framing, Development of people and skills, Aspirations and Framework feedback, were subsequently generated through the application of metacognition scaffolding technique (Jumaat and Tasir 2014) which stimulated participants to reflect on their experiences, understandings and learnings during their session(s). Parent and child node frequencies are shown in Table 3.

**Table 3:** Parent and Child Node Frequencies from QSR-NVivo

Parent node	Child node	Frequency	Percentage (%)
Attitude		148	14.77
	Technology	20	
	People	128	
Organisational Subjective Norms		202	20.16
Capability		263	26.25
	Organisational Technological Capability	63	
	People and Skills	200	
Framing		46	4.59
Development of People and Skills		52	5.19
Aspirations		39	3.89
Framework Feedback		252	25.15
	Endorsements	129	
	Potentials	123	
Total theme frequencies		1002	100

Participant details are shown in Table 1. For the purposes of anonymisation, participant genders were changed to ‘[[she/he/they]]’ and in some parts of transcripts changed to ‘she’ when referring to others. The job titles held by participants were anonymised through equivalating titles to similar positions. Throughout this article participants are referred through the following system: pseudonym initials (as presented in Table 1), company number and designation seniority. For example, participants from company reference 1 are referred to as “AL:1:1” and “GL:1:2”. Similarly, participants from company reference 5 are referred to as “MK:5:1”, “ST:5:2” and “DS:5:3”.

### Attitude: people are a strong defence, but technology is a known friend

Participants’ attitudes were captured prior to and during their session(s) as discussed in the section above. Attitudes reflected participants’ beliefs regarding ‘technology’ and ‘people’ in the context of UIT and cybersecurity more widely.

#### Technology: the main contender for defences despite known limitations and unequal application

Analysis from the qualitative data showed a lack of confidence in certain technological defences and techniques such as those pertaining to encryption and phishing. Historically, a technical defence, such as encryption, has enjoyed immense popularity and extensive application in industry for end-use products taken to market. Similarly, phishing simulations received mixed and often contrary guidance from regulators. This is reflected in the comment below,

‘We did look at doing simulated [phishing attacks] but actually I pulled back on some of the effort we were putting into this because [[UK government]] doesn’t advise on attack simulation in their latest guidance’ - BP:2:1

From discussions, all participants were confident in their understanding of technological defences and their organisational readiness levels (whether weak or strong). Participants also reflected an attitude of prioritising some assets and systems over others:

‘There’s also tiers of that as well. So, I mean, the stuff that [[team name]] [does] is the critical vulnerability stop, so we [act as] soon [as we] hear about that. There’s also the less critical stuff, updates and things’ - DS:5:3

Consequently, some systems and assets, usually newer ones, were better protected than others resulting in an unequal application of defences across utilised technologies. Participants’ attitudes also showed a preference for using technology as opposed to humans to create and maintain defences:

‘One thing you might not be aware of [[ST]], is that we do quite in-depth penetration test and [[exercise name]] that does establish some understanding of our hygiene. So, because it’s quite a [[controlled]] environment, we take the approach of talking to the computers rather than the individuals’- MK:5:1

#### People: there is strong faith in others but against the backdrop of human fallibility

Data from quantitative questions reflected a change in attitude after using the tool in the context of increased belief in UIT levels being static over short periods of time (shown in Table 2). Participants’ attitudes also appeared to positively shift away from believing that UIT arose as a direct consequence of users deviating from prescribed processes. Qualitative data showed participants having trust in other people’s skills due to close-knit relationship structures that existed within their organisations, e.g.

‘On the other end of the scale, they [users] do feel that everyone’s there, ready [to help]. That it’s not just them, there’s a bunch of people [to help]. I’m not going to go into process because we’ve just done processes [input] but there’s at least lots of other people around who can help’ - MM:3:1

This structure was reflected through discussions around strong peer-to-peer support being available, informal training being offered, known individual skillsets and knowledge sharing that occurred within small teams at large organisations or within smaller organisations. Overall, however, participants demonstrated low confidence in the effectiveness of formalised and external training programmes for themselves and others.

Our dataset also showed mixed attitudes towards communications. Participants believed people possessed good communication skills when communicating with each other and contributing openly and effectively towards enhancing and developing processes. However, participants showed a negative attitude towards people oversharing or communicating negative information (such as feedback pertaining to flaws, limitations or challenging processes, ideas or technologies). For instance, the following exchange between two participants:

‘I think we should change that [input] to ‘users will, ‘without fail’, report processes that they consider to be inaccurate’ [laughter] (KK: In this organisation definitely) [laughter]’ - GH:6:1; KK:6:2

Participants also showed a positive attitude towards empowering others, evidenced in the following dialogue between two participants:

‘HR: I’m not talking about the implementation yet, right, but (MM: Learning) we didn’t stigmatize anybody for SolarWinds (MM: Right) or [[another example]] or anything else. So, from that perspective, we spent a hell lot of time to look at what gets better. And now, since we’ve had so many of those, I don’t think its platinum [level 4] yet because it’s not across the whole organisation with everybody but it’s [getting to a learning culture]. I would argue it’s certainly gold [level 3].

MM: Yeah. And I think in some cases, I’m sure there’s stuff going on where people think, ‘I’d rather not say anything’ but, I think that’s more the exception now’ - HR:3:2; MM:3:1

This empowerment was also evidenced in discussions that emphasised others learning, not stigmatising mistakes, being able to ask questions to understand wider strategic objectives, not having reprimands as consequences and being able to solicit the right people for advice. There was also a positive attitude amongst participants of being understanding towards others when discussing the occurrence of mistakes, unintentional errors, lack of experience or lack of knowledge:

‘I wouldn’t necessarily say, depending on what’s going awry, [that] everyone knows how to protect themselves and thereby, sits by using the standard safeguards that are already on the system’ - ST:5:2

### Organisational subjective norms: continuous improvement

Data within this theme reflected participants’ subjective norms. In line with the Theory of Planned Behaviour, data codes revealed that subjective norms amongst our participants constituted of two aspects: normative beliefs within their organisations and, their motivation to comply – both of which were shaped by others around them.

All responses within the questionnaire pertaining to this theme showed a change in participants’ ratings prior to and post session(s). After using the web-based assessment tool, participants showed a greater inclination for sharing knowledge about cybersecurity related practices and near-miss experiences across their organisations. Whilst participants initially indicated that it was “everyone’s responsibility” at the organisation to be aware of cybersecurity challenges, they did not subsequently indicate a wider organisational interest for insights gained during the qualitative session(s). Participants’ subjective norms indicated that they intended to take action to strengthen certain defences identified in the personalised report which was in line with participants’ earlier indication that everyone was able to act if a cybersecurity vulnerability was identified. In contrast, participants also indicated that it was expected of them to be responsible for organisational cybersecurity by others, and senior management would support their initiatives to strengthen their organisational cyber defences. Prior to session(s) participants indicated a strong inclination for considering workload, procedures and resources at their organisations when creating cybersecurity practices which was reflective of their subjective norms. This sentiment showed an increase in inclination following the use of the web-based assessment tool.

From qualitative data it was evident that participants subjective norms reflected feelings of prestige associated to their organisations:

‘Susceptibility [laughter] Because like […] we rank everyone [other organisations] as well’ - RR:4:1

This positioning meant that participants viewed their organisations as leaders within specialised sectors. Subsequently, participants’ social norms indicated upholding high standards for conduct, practices and delivery, with little room for errors. Participants enjoyed a shared sense of pride associated to organisational prestige and indicated a social norm for active risk awareness (such as reputational risks) which resulted from this organisational positioning.

Participant discussions during semi-structured interviews revealed that certain parts of the organisation (departments and/or processes) were believed to be performing better than others. This understanding meant that organisations were able to critically appreciate their areas of strength and limitations. For instance,

‘I think it’s because the risk is low [data asset value or penalties] and maybe this is the reason why it is a lower rating for [[department name]]. Because the data that that [department] holds, isn’t classified as “sensitive data”. Whereas you mentioned [[my department]], obviously we hold sensitive data and therefore it’s better protected (BP: Yeah) So it’s all about proportionate risk, (BP: Yeah). I’m still of the opinion that for the nature of the business, the amount of data, the type of data that we hold, we’re doing enough [laughter]’ - SN:2:2

Participants’ subjective norms evidenced an organisational focus to discover, understand and improve mutually understood limitations:

‘We have too many processes and not all of them are fit for purpose. That may be harsh, but we need to improve our processes and that’s for sure (RR: Right) And that [weakness in processes] is shining through here [in the personalised report]’ - KL 4:2

Furthermore, participants’ context was framed by their organisational composition of highly skilled people within highly specialised industry sectors. This context appeared to aid participants in gaining a deep understanding of their organisational strengths and limitations against UIT. For instance,

‘We are a [[specialised industry sector]] company and a lot of our staff or, well the majority, would have advanced skills rather than just basics. So definitely not surprised there [for being on level 4]. ‘In-house IT skills’ [output] that also [is high expertise]. Yeah, we’re a skilled department’ - SN:2:2

Participants subjective norms indicated an active knowledge sharing culture amongst people at their organisations. For instance, the following quote from a participant when discussing near-misses, communicating them widely within the organisation and utilising them as learning opportunities,

‘I think we’re very good at that [learning from near-misses]. We have quite an open culture basically and if we’re talking about cybersecurity near-misses here, if there has been a cockup and, there have been the odd one or two, then we definitely don’t hide those. They’re definitely widely discussed’ - AL:1:1

Knowledge sharing occurred internally and externally through formal and informal channels whereby people could also challenge and question information that was being provided to them. For instance,

‘I’d say most people are quite happy to question (ST: Yeah; MK: It’s what we do) [...] ‘Question’ yeah, ‘share’ yeah, ‘challenge’ yep, absolutely’ - DS:5:3

In some instances, subjective norms indicated that organisations would decide the nature and extent of knowledge being shared with others. However, this choice did not appear to be driven by malice but rather a need to safeguard people (for instance, from feeling overwhelmed).

Subjective norms amongst participants reflected an attitude of accepting human fallibility with a shared sense of culpability within the organisation. Expecting and accepting errors stemmed from participants accepting imperfection within themselves, and working with imperfect systems whilst meeting organisational demands. This is reflected in the quote below:

‘Because the thing that makes me think about [factors] is that actually people are more susceptible to make mistakes when they are overloaded, and when there’s not enough capacity in the organisation. We all do it. And I’m going to do it sooner or later. And you know, that’s ‘me’. I should know better. And there will be people within the organisation who, because they’re very busy, click on something they shouldn’t do’ - AL:1:1

Participants’ subjective norms indicated that overall processes were viewed to be effective, people were able to effectively multitask, prioritise and manage workloads. People were also able to give feedback and feed into the revision and creation of organisational processes. Subjective norms within this theme indicated that people could openly communicate and report processes that were inefficient. However, participants’ discussions reflected a tension between processes, workload and capacity and the overarching organisational need for growth and maximized delivery, as demonstrated in the quote below:

‘Workload is only ever increasing. Even if we just do the same [amount of current projects]. But we want to grow, right? So, we’re going to be constantly at a point where we have to balance employee well-being, which is our first priority, and then growth scenarios. And at times, it’s just going to be a stretch’ - HR:3:2

Amongst all our participants subjective norms indicated strong relationships between people laterally at their designations and vertically with others. This meant that people were able to ask for and receive help through formal or informal channels. However, GH:6:1 went on to state that due to a perceived blame culture these strong relationships also meant that people were able to delegate responsibility to others at higher designations:

‘We have a real problem in the organisation with upward delegation. So, yeah, ‘I’m just going to delegate that task, that responsibility, that decision making to somebody more senior than me’, and it happens an awful lot of in the organisation […] Now, that comes partly, although we kind of circumnavigated a bit, there is a bit of a blame culture in the organisation that is hypothetical [placebo] […] And that’s something we’re trying quite hard to break’ - GH:6:1

Participants also indicated a subjective norm of enjoying support from senior management, such as Board Members, when undertaking cybersecurity initiatives at their organisations.

Finally, participants indicated that defences were stronger than they had expected prior to their session(s). For instance,

‘What I take away from this [report] is probably [the organisation has] more robust set of processes, having thought about it [while] going through the questions, than I would necessarily have said straight up [prior to interview]. But what I take away from it is that means that we are in a position to probably better hone in on the outliers without then having to think we have to eat the whole thing at once’ - MM:3:1

Lower expectations amongst our participants reflected a subjective norm of underestimating the strength of defences in place and organisations feeling more vulnerable than perhaps they are in reality.

### Capability: variance in perceived control

Capability theme reflected participants’ perceived control through the availability of opportunities, resources and self-efficacy in two primary contexts: technologies and people. Results indicated that participants believed to have strong perceived control at an individual level when dealing with others (as others were seen to be individually competent) and through the effective use of technologies. However, this perceived control was weak when discussing people as groups and over the availability of resources at their organisations.

Quantitative analysis indicated participants were more inclined towards a shared sense of responsibility for cybersecurity within their organisations i.e. a distributed levels of control following their session(s). Participants indicated control over the design of procedures and processes (theoretically and in practice) and responses reflected a positive correlation between procedures/practices and cybersecurity – with all ratings towards ‘Strongly Agree’. Participants initially indicated influence over those around them (such as being able to start working groups and communicate ideas and findings to board members). However, following their session(s) this perceived control did not appear to permeate into practice. Finally, participants expressed increased inclination of control through technologies such as restricting user access and privileges in IT systems to limit UIT following their session(s). This is discussed in greater depth in the following sub-sections.

#### Organisational technological capability: effective use of technologies equates to increased feelings of being in control

Participants had a high level of perceived control for avoiding UIT through organisational technological capabilities. Participant discussions during semi-structured interviews reflected good organisational technological capabilities which acted as passive cyber defences for instance, good configuration, ability to monitor users and devices, managing user privileges and ensuring confidentiality of data. Participants shared that they were able to consider existing technologies prior to implementing new ones and were aware of their technical capabilities (strengths and limitations) during their session(s). This contributed to participants’ having a high level of trust in technologies, self-efficacy and a strong perception of control through technologies to circumvent UIT. This is reflected in the conversation between two participants below:

‘We operate the system of ‘least privilege’ where we can, [with]in the organisation. It’s highly unlikely that we will have a system where every user is an administrator of that system, every user is a power user etc. So, when it comes to how system access is organized, yes it’s a known, auditable, laid out thing […] We do also know who the ‘super users’ [users with increased user privileges] are (KK: Yeah) So, that side of it, yes, it is known, audited and documented (KK: And we share it as well) yeah, exactly’ - GH:6:1; KK:6:2

In contrast, participants depicted lower levels of perceived control for data management as it involved human ways of working which meant it was believed to be more challenging for participants to control, for instance,

‘Because there’s some unstructured data that’s copied in [places] and we’ve got a tool that look kind of looks at that. But we’re very confident about that [level of data duplication]’ - RR:4:1

#### People and skills: higher perceived control on an individual scale with lower levels of control over groups and organisational resources

Semi-structured discussions reflected high self-efficacy through exhibiting strong confidence in others’ capabilities. This strong belief in others often circumvented the need for formalised training programmes that could be offered by the organisation, for instance,

‘The emphasis on formal training [laughter] I think to an extent it doesn’t take account of the fact that when we are quite a small company, we can achieve an awful lot without some of the formal training’ - AL:1:1

Capabilities included a range of aspects. It comprised of others’ ability towards technologies for instance, proficient use, being able to identify malicious content and spot inconsistencies in software. It also included others’ ability to be proactive and also take action if things were amiss. Furthermore, participants indicated strong belief in others being able to question or challenge concepts, practices and guidelines. Participants believed that people practiced procedures and actively took an interest in learning new things. Participants reflected strong faith in others being able to effectively prioritise and be able to identify and request help and advice when needed. Participants also reflected a critical understanding of processes that were in place at their organisations which included the knowledge of processes that were effective or ineffective and those that needed further improvements. This is evidenced in the conversation between participants DS:5:3 and ST:5:2,

‘DS: Yeah it’s that weird [[specialised industry sectors]] thing, isn’t it? Actually, [[specialised]] staff in particular are quite vocal on that sort of thing [processes] (ST: Mm) that they do proactively feedback and (ST: Yeah) it’s one of those things.

ST: I mean I think it’s a mix. The thing is that ‘experienced users feel prescribed processes are effective’ well, not necessarily, but they do report inefficient things’ - DS:5:3; ST:5:2

However, participants showed low confidence in collective capabilities of people i.e. they were confident in a majority of people being highly competent but not *everyone* within their organisations. Thus, participants were mindful about the variance in capability whereby a few outliers could compromise the overall security and integrity of their organisational defences. This favourable evaluation on an individual scale can be representative of a person-positivity bias whereby aggregated favourable values are less appealing when represented as groups (Sears 1983). This is reflected in excerpt from the conversation with KL:4:2 below:

‘So [[company name]] has a lot of technically able people, but it also has a number that probably aren’t IT experts in certain fields. And you’re going to have that in an organisation. We’re not all Google, it’s not like Google where everything is IT. So I think that’s quite interesting because we’re relying on people’s ICT skills against insider threat vulnerabilities’ - KL:4:2

Overall, discussions reflected low self-efficacy and control over people’s capacity and availability of resources at participating organisations. Participants also indicated lesser control over senior stakeholders such as board members due to time constraints and communicating in ways that resonated with them. Participant KK:6:2 discussed this challenge by sharing,

‘For me, it’s usually the ‘high end users’ [senior stakeholders] as I would call them [laughter] that are problematic for us in terms of implementing new security measures. As [[GH]] said, that [change] would involve some systems being less flexible, or less convenient. And this is what triggers users usually. Especially if we haven’t got anything to say like, ‘We’re putting this [change] in place because someone has hacked [us] or someone actually did this’. If we’re saying to them [that we’re implementing changes] because ‘We think there is a real danger currently out there and we should protect ourselves and we should protect the organisation’, they [users] don’t see it as the most important [priority] thing that we should apply it [implement change]. So that’s the challenge we’re dealing with’ – KK:6:2

### Framing: organisations deliver a mixture of various services

Participants viewed their organisations as dynamic entities which existed in complex environments:

‘Sometimes there are still tasks allocated if you haven’t, it’s really down to interpretation, right? If you think about, if you are in an incident response team, you will get a task allocated at short notice (MM: Always. Always) this is the business. So, the question is, is the capacity enough to always deal with that. And since it’s part of that business process, you know, you just have to have staff and avenge to deal with the impact. Versus, programmed [planned] business, you know day-to-day operations, which tends to be different [steadier]’ - HR:3:2

Consequently, when interacting with the web-based assessment tool, participants considered various aspects of the business prior to selecting the best suited readiness level for their organisation. Aspects through which participants positioned their organisation entailed considering various internal or external systems. ‘Systems’ included software, processes, workload, skills, stakeholders and the nature of service being delivered. Systems’ legacy within the organisation (in processes and equipment), geographical location and size were also aspects taken into consideration by participants. This meant that organisations were believed to exist in mixed states simultaneously for readiness levels against UIT. For instance, this system complexity is showcased in the quote below,

‘I think the other thing to add is [[organisation name]] is quite unique in how it’s structured. So, I don’t know [[IV]] if you know, so as we said, [[RR]] and I work in a group function. But we [also] have the individual business units, [[independent, autonomous domains]] or however you want to call them. So, they [independent domains] have their own operates and processes in themselves or [they] own certain processes. So it’s quite a complex environment’ - KL:4:2

### Development of people and skills: there is a commitment to upskill people through in-house resources within functions they perform for the organisation

Discussions reflected strong, informal structures between people at organisations participating in this study. Organisations were believed to provide ‘guided training’ and development to their employees. Participant discussions exhibited team reliance for support and knowledge sharing, informal channels of communications, mentoring and teaching expert skills to others, and contributing to processes through feedback and reporting. Guided training meant that people were developed and up-skilled in more informal ways such as in-house training, inductions, on-boarding, on-the-job training, handbooks and other forms of organisational communication channels:

‘Clear example here, the entire technical team we’ve just been through [[writing safe code]] training (GL: Yeah) because it is outside their skills at the minute. There’s a lot of training that we don’t offer in-house, so we did lots of external stuff’, AL:1:1.

Whilst training was offered by organisations, a specific training programme was only provided to individuals if it was viewed as being directly relevant to their job function within the organisation. While communications were used to upskill and develop people, less willingness was shown by participating organisations to share information fully and transparently with everyone within the organisation.

### Aspirations: consistently improving as a goal

Participants shared organisational aspirations to improve various technical and sociotechnical aspects, for e.g. by saying,

‘One of the consequences of that will be that there are areas of data management that we could improve […] But it’s nevertheless something that we could do better at’ - BP:2:1

And,

‘After seeing the questions and then analysing how our users will react, I think this is something that we’ll definitely make users more aware of. And put much more effort into user guidance and user awareness of what is cybersecurity, what’s social engineering and all the other stuff. So they [users] actually know what to expect and know how to report this to us and not to be scared of reporting this [anomalies] to us because this is a very important bit [factor]’ - KK:6:2

Organisational technical ambitions were framed around improving elements such as encryption, software architecture, data management, asset protection and network security. Organisational sociotechnical ambitions included improving people’s knowledge, growth, relationships, processes, management of workloads and capacity and communications. For instance, MM:5:1 shared aspirations to improve others’ learning and communications following the session(s):

‘Yes, I can see that now, horizontally and vertically people would communicate, culture is going to be relatively appropriate, we’ve got at a medium level of protocols, but I can see that we could do more on the ‘learnings’ and ‘communications’ [aspects]’ - MM:5:1

### Framework feedback: elements that worked and those that could be better

Throughout their session(s), participants shared their thoughts about the factors being considered, the web-based assessment tool itself, the generated personalised report and their experience of the session(s). Overall, discussions pertaining to these elements encompassed two themes: endorsements, and potential improvements which included limitations and recommendations for the assessment tool.

#### Endorsements

Participants found their session(s) to be a positive experience:

‘MM:3:1: Certainly made me think. I was a little concerned actually thinking, ‘Oh my goodness, three hours of this thing’ but it’s certainly something that made me think. And you [IV] and I have discussed stuff which we don’t in a normal day-to-day of things, we don’t take some time out to do things. So, I’ve certainly enjoyed it.

HR: Yeah me too. It turned out to be quite fun, actually. I thoroughly enjoyed it. I would do it again [laughter] with different questions maybe? [laughter]’ – MM:3:1; HR3:2

And,

‘GH:6:1: I quite liked the challenge of being made to think about things - things that I wouldn’t necessarily have thought about before. Or things being presented in a different way. So I think that would be very much around the fluctuating vulnerabilities [pillar and factors which] was the key one for me, thinking through that. And also, I don’t know how to put this, [but] we do various things, we know what we do and we will present that as ‘[[organisation]] does this, this and this’. Done. Boxes ticked everywhere. [When] Somebody asks you a question that doesn’t quite fit into the way that you do stuff, [then] you have to really think about what you do. So, I actually found that quite fascinating. Having to think about things, when it’s somebody else that’s asking me the question actually, was really helpful. And also to my mind, I think it was a little bit away from [typical assessments] as we do get audited quite a lot. At least three times a year we have to fill the forms in for our auditors. This [assessment tool] asked very different questions to be honest.

KK: [It’s a] Different point of view. (GH: Yeah) Yeah’ – GH:6:2; KK:6:2

The assessment tool and the session(s) appeared to elicit reflection amongst participants and reimagine their current approaches. Session(s) allowed participants dedicated time to evaluate their UIT defences in isolation to the wider cybersecurity defences and the presentation of the web-based assessment tool afforded them the ability to examine factors in an unconventional way i.e. by repositioning the way humans are understood within systems in the context of safety and security.

Due to the application of metacognition scaffolding technique participants were asked to reflect on their experience. Participants reported gaining new insights through their experience of interacting with factors being considered through the web-based assessment tool and the personalised organisational report. Participants reported that their session(s) allowed them to think differently from the currently established ways of evaluating defences. Additionally, participants shared intentions to adopt a human-centric perception (including through acting in new directions) in the future, for instance a quote by BP:2:1 below. From discussions during the sessions (between the participants themselves or with IV), it was evidenced that new knowledge was acquired, or existing thoughts were reinforced about factors that interact with UIT.

‘This [factor] is perhaps one for us to reflect on. It is a question of workload and whether there’s a risk that sits behind that, that we need to spend a little bit more time thinking about as a business. [...] I think this [output] is probably something that we could call out as a as a clear take away [insight] for us. It’s probably a new risk for us to capture in our risk bank’ - BP:2:1

Participants described the outputs within the personalised report as being in-line with their expectations for strengths and weaknesses of various organisational defences that were being evaluated:

‘To be honest, that [personalised report outputs] is about right for me (GL: Hmm) Because actually, fundamentally, we are a [[industry sector]] company and we advise other companies on their software architecture. This actually tells me, I think it’s a fairly realistic picture, that we’re very good at the architecture, very good at identifying what should be going on, very good at keeping on top of the basics. We’re slightly less good at the process driven stuff and so monitoring, data management and that stuff which interestingly is the human factor’ - AL:1:1

Additionally, participants shared that the personalised report effectively highlighted areas that needed continued organisational attention, areas that need improvement, and aspects that might contain weaknesses within defences in the near future (for instance due to organisational growth). Discussions also reflected that participants discovered new factors to include in their line of defences against UIT. For instance the quote below from a discussion between BP:2:1 and SN:2:2. Overall, participants shared that the findings contained within the organisational personalised report were relevant and accurate.

‘BP: SN, I can’t remember going through any of the processes we’ve been through recently [ISO 27001] where this [workload] was a discussion point [highlight] for us. I’m not even sure whether we have this risk captured in our risk register?

SN: No, I don’t think so. I don’t think I have, no. I don’t think I’ve seen this [workload being considered] before. So [this input is] one of those notes [for us to think about] workload and resources

BP: Yeah. I think this [output] is probably a new a new risk for us to capture in our risk bank’ - BP:2:1; SN:2:2

#### Potential improvements

For various elements within the web-based assessment tool, baseline measures for adequate technical readiness levels were believed to be too high for many organisations. Baseline measures were developed through recommendations by NCSC, CERT and previous research by Khan et al (2021). While these levels are believed to be the minimum recommended level of technical defences for organisations, our data indicated that either certain parts of an organisation or, organisations overall, would be unable to achieve said levels. This is reflected in following comment,

‘Just as an observation on the questions, some of the [technical levels], I wonder if it might be useful to have something below bronze [level 1 on the assessment tool]. Because actually in some cases bronze [level 1] is actually a reasonable bar for some organisations. What you might find is that as you’re answering the questions you think, ‘Oh, we’re probably somewhere in the middle of between bad and good’. But actually, when you read the writing, sometimes the worst option is still relatively good. So, for encryption [input], I can think of some organisations that haven’t got that high priority data assets encrypted yet. Offering that question, offering a level below bronze, might be useful to get more accurate answers’ – MK:5:1

Participant discussions reflected their recognition of elements from established frameworks and guidance that were incorporated into the web-based assessment tool. Recommendations from participants included capturing additional technical elements and the use of machine learning algorithm to inform the personalised organisational report and analysis.

Input from human factors and computer science domain experts aided in creating descriptors for each level within the assessment tool which aimed to be subtle to support ‘outside-the-box thinking’ and to limit the influence of any pre-established notions, beliefs and perceptions amongst participants. However, participants sometimes expressed difficulty in selecting an appropriate rating due to the subtle differences in meanings and expressions reflected within each of the levels. Some participants indicated a need for having levels that are singularly focused on an element within its broader category and increased choice for options that went beyond ‘don’t know’ and ‘not applicable’ if none of the levels appeared to be an accurate representation of the organisation’s current state of defences. Participants also made recommendations for the framework to recognise different training techniques that were believed to achieve similar results to formalised trainings, such as those discussed above (Sect. 4.5). Such training and development techniques (for instance formalised training programmes being replaced with in-house training, on-the-job training, mentoring etc.) are likely to be utilised by specialised sectors or SMEs. Following the Covid-19 pandemic, participants also indicated the importance of including and evaluating defences that are reflective of flexible, hybrid and remote working as part of the framework and assessment tool. This is evidenced in the quote below:

‘I wondered whether you [IV] would consider as part of this data capture is where people are working. To [address] the point around in-house ICT skills, that can often be compensated in some non-technical environments by community knowledge. Like asking the person in the next cube [desk] to you what they do, who then asks [someone else], you know what I mean? That’s a lot more difficult to orchestrate when working remotely on Teams. I wonder whether that [remote environment] is a factor in determining in-house ICT skills, is that a problem or isn’t it? In reality, when you overlay [map out] how people tend to work, then you can understand that, actually, if they’re remoted [remote working], the ability for them to reach over the cube [desk] and say, ‘Mm, have you seen this before?’ is diminished (KL: Yeah) and we’ve seen attacks leveraging that kind of social construct’ - RR:4:1

## Discussion

Guided by the Theory of Planned Behaviour (TPB), findings discussed above reflected participants’ attitudes, organisational subjective norms, and participants perceived control over unintentional insider threat (UIT) at their organisation. Metacognition cognition scaffolding technique provided additional findings to TPB i.e. organisational framing, function-related development of people and skills, and organisational aspirations to improve cybersecurity defences.

The web-based assessment tool derived from the framework from authors’ earlier work fostered reflection amongst participants, and attitudes towards UIT from a human-centric positioning noted a positive change on the quantitative measures prior to and post session(s). For instance, participant attitudes moved away from believing that UIT was a result of individuals deviating from prescribed processes, demonstrated higher confidence levels for understanding their organisational sociotechnological defences (strengths and weaknesses), and participants showed a decline in attitude towards popular techniques that have been widely implemented (e.g. phishing simulations and encryption). Despite an inconsistent approach towards the application of technological defences to safeguard assets within organisations, participants retained a favourable attitude towards technology – to create, maintain and evaluate defences to safeguard against UIT. These attitudes are potentially reflective of, and intertwined with, the wider societal view that favours technology centric solutions stemming from traditional security thought, for their perceived ease in building and maintaining defences to circumvent insider threat.

Qualitative discussions with participant discussions reflected a positive attitude towards others to create strong lines of defence against UIT. This attitude was centred on knowing others’ skill level, having faith in others’ ability and the existence of strong interpersonal relationships. It was further reinforced through peer-to-peer support networks, informal training programmes, knowledge sharing, and learning and empowerment that occurred at an individual level. Oversharing or inappropriate communication skills, effectiveness of formalised training programmes and human fallibility at individual level limited the positive attitude demonstrated towards others. Human fallibility taken into account by participants appeared to be at a surface level which was understood as an inevitable human condition. Deeper qualitative discussions did not provide evidence of deeper understandings amongst participants for the type of errors that occurred i.e. slips, lapses, mistakes and, violations (Reason 1990), nor the type of cognitive tasks that resulted in errors i.e. actions that reference Skills-Rules-Knowledge based actions (Rasmussen 1983). Discussions reflected participants’ understandings were acquired from some of the elements presented in MERIT (i.e. known individual skillsets and opportunities afforded to individuals) and error management programme (i.e. knowledge sharing, empowerment and learning). Research conducted by Noble (2018) found managerial favourability towards technological defences to safeguard cybersecurity vulnerabilities with a reluctance to consider human factors. While our research study found that there was still a preference for technological defences, participants exhibited a positive attitude and understanding of human factors that interplay with UIT.

In line with TPB, participants’ positive attitude towards technology and individuals might indicate the ability to effectively strengthen defences against UIT. Kabanda et al. (2018)’s findings suggest that attitude plays a role in the effective implementation of cybersecurity at organisations. In another study (Hadlington 2017), positive attitudes to cybersecurity at work can lower risky online behaviours in people’s personal lives. The same study also reported ‘non-planning’ as a significant predictor of risky cybersecurity behaviour. Within this lens of TPB, our findings show that participants’ positive attitude, both towards technologies and human aspects, would be desirable when implementing change to strengthen organisational cyber defences against UIT.

Following their sessions, data showed an increased inclination amongst participants to share knowledge (i.e. near-misses and best practices) more widely within their organisations and to implement changes that can potentially strength defences against UIT. This inclination showcased participants’ subjective norm belief of everyone being responsible for cybersecurity at participating organisations within this study. Additional findings for subjective norms included support from senior stakeholders and, responsibility and accountability from senior personnel for cybersecurity related aspects. Huang and Pearlson (2019), highlight the importance of senior leadership taking special responsibility to meet organisational cybersecurity goals – an aspect that was reflected in participant discussions pertaining to the subjective norms within their respective organisations. Subjective norms reflected in participant discussions appeared to consider workload, procedures and resources when designing cybersecurity defences.

Subjective norms also included upholding high standards i.e. personal conduct, delivery, practises, risk awareness and feelings of pride linked to organisational prestige and low margins for error. These reported subjective norms would in turn serve as motivation for people to comply with procedures set out by participating organisations. Participants’ subjective norms were demonstrated in their critical understanding of the strength of their UIT defences which included a desire to discover, understand and improve factors that affect defences. These subjective norms were in conjunction to a normative belief of sharing knowledge with others, as long as such sharing did not negatively impact audiences. This stance on knowledge supports ‘effective communications’ within the recommendations by NCSC (2012) to create effective cyber defences.

Individual attitudes of accepting human fallibility discussed above appeared to be reinforced by participants’ subjective norms. However, human fallibility as a subjective norm was linked to organisational demands and imperfect systems used to deliver tasks. This finding provides evidence for CERT (2013) framework which associates UIT to factors such as time pressures, task difficulty and cognitive load on individuals. Participants shared a normative belief that their organisations implemented effective processes. Effective processes meant that participants believed that people were able to inform organisational processes, efficiently multitask and manage assigned workloads which substantiates findings from the error management programme (Liginlal et al. 2009). Normative beliefs also included strong peer support being available within organisations and an underestimation of the strength of cybersecurity defences in place. Additionally, participants’ subjective norms denoted an understanding of the tension between delivery of work (processes, workload, capacity) and an organisational desire for growth and maximised outputs, which in turn can adversely effect defences against UIT. This tension between people delivering tasks and organisational context (Fig. 3; Khan et al. 2021) as a subjective norm can potentially penetrate otherwise strong defences and bolsters the need for a human centric approach to understanding the varying strength of UIT.

Overall, these subjective norms at participating organisations within this study reflect a preliminary, sociotechnical foundation in place which can foster stronger defences against UIT. This foundation can be further built upon with interventions, such as the web-based assessment tool, to create and maintain effective sociotechnical defences over time.

Participants shared an inclination towards distributed levels of control to maintain strong cybersecurity defences. Amongst participants there appeared to be high perceived control over the design of processes and procedures, both aspects that participants believed influenced the strength of defences. Thus, participants perceived to have control over defences through their ability to design and influence processes and procedures at their organisations.

High perceived control was also depicted amongst participants to prevent UIT through the use of technologies. In fact, all participating organisations reportedly had strong technological capabilities, with a mixture of passive and active cyber defences already in place. The implementation and use of active and passive defences are supported by NCSC (2012), CERT (2006, 2007, 2008) and Nurse et al. (2014). Findings reflected that participating organisations considered existing technologies prior to implementing new ones—providing evidence for Liginlal et al.’s earlier findings (2009). Data management was the only technological element with a reduced level of perceived control as it involved others (i.e. humans). Overall, participants indicated high levels of perceived control, self-efficacy and trust through the use of technologies to create strong defences. The heightened perceived control through technologies that is expressed by participants can also be a significant contributor to the preference for technologies by senior management as suggested in the findings by Noble (2018).

Similar to control through technologies, our findings revealed that participants believed others’ skill on an individual level contributed to their own self-efficacy and control over defending against UIT. Participants believed in others’ individual ability to actively contribute to, practice and challenge processes, continuously learn, ask for help and solicit advice, question and challenge concepts, effectively prioritise, possess effective technological capabilities, be proactive and take action to correct course if something was awry. This strong belief in others’ ability at an individual level which contributed to their own self-efficacy and control was at times noted to come at the cost of diminished belief in formalised training programmes.

However, participants exhibited lower levels of perceived control over the availability of resources and capacity within their organisations and when people are represented as groups. Resources comprised of the availability of people (i.e. linked to turn-over) and time available to deliver outcomes. Capacity included people’s ability and availability to perform functions (i.e. associated to workload and cognitive loads). Participants also demonstrated lower levels of control over their ability to defend against UIT when discussing people as groups for instance, when groups of people were represented as departments, teams or at specific job designations. Similarly, when discussing senior stakeholder groups participants shared sentiments of reduced control which stemmed from factors such as time constraints and finding effective ways of communication. Subsequently, the perceived variance in skillsets emerging from collective capabilities translated to lower levels of perceived control amongst participants.

Overall, these findings for perceived control indicate that participants possessed high perceptions of control which is exercised through the effective use of technologies and self-efficacy which is achieved through the ability of others at an individual level. However, participants indicated lower levels of perceived control over resources, capacity and groups of people. This can potentially indicate that whilst participants believe to have control through utilising technologies and others’ ability, this perception of control is diminished when faced with the larger systems that exist within organisations.

In the context of TPB overall, participants demonstrated a positive attitude towards technologies and people which could assist participants in building and strengthening their organisational cyber defences. Additionally, existing subjective norms and motivations to comply are favourable for participants in their current efforts and might provide advantageous conditions for them to innovate new defences in the future. Whilst participants demonstrated high perceived control through technologies and individuals, their control was limited when interacting with larger organisational systems. Thus, TPB suggests participants would encounter challenges when faced with devising, implementing or strengthening organisational wide defences against UIT.

Findings show that organisations need to position or frame themselves in a distinct way when assessing their cyber defences. Considerations paid to ascertain organisational positioning when conducting assessments can include a range of aspects such as implemented software, processes, workload, stakeholders, geographical location, and size of operation. Consequently, the outcomes depicted as part of any assessment tool will only be accurate in the context of the organisational position chosen by those involved in the process.

Session(s) afforded participants an opportunity to reflect and introspect, fostering a change in thinking and gaining new perspectives about established challenges associated to UIT. The aspects considered were reportedly different from standardised assessments (such as ISOs) and participants described gaining insights to strengthen their defences. Outputs were deemed to be an accurate representation of current defences, effective in highlighting strength of defences and identifying potential areas of concern. Additionally, participants reported discovering new vectors that can be included as part of their existing UIT defences. The web-based assessment tool’s categories for readiness levels can be enhanced further to become more varied, include additional technical aspects (e.g. ISO 27001; Brenner (2007) 27001), and inputs can be made more distinct whilst offering new options alongside ‘don’t know and ‘not applicable’. Varied training techniques can also be incorporated into the framework and inputs can be reflective of hybrid/remote working implemented post the Covid-19 pandemic.

## Conclusions, limitations and future work

A web-based assessment tool was created from the framework proposed in earlier work by the authors (Khan et al. 2021) which captured 45 distinct vectors displayed across six pillars to determine the strength of organisational cyber defences against UIT through a personalised report.

Guided by the Theory of Planned Behaviour (TPB) a mixed methods research study was designed to investigate the impact of framework on strengthen defences against UIT. Thirteen participants from six organisations were recruited through National Cyber Security Centre’s ‘industry 100’ partners and the first author’s professional contacts. Participants represented senior and mid-level leadership roles within various sized organisations (SMEs, large and non-profit). Participant’ attitudes were measured through semantic scales prior and post session(s), capturing discussions whilst they interacted with the assessment tool, and through semi-structured interviews as this was believed to be indicative of session’s influence on their planned behaviour towards their cyber defences. Template analysis approach was applied to code approximately 14 h and 30 min of qualitative data to uncover findings.

The session(s) appeared to elicit reflection amongst participants which allowed them to gain new perspectives to established UIT challenges and added new vectors for them to consider as part of their existing defences. Participants also shared gaining insights from generated outputs. Overall, participating organisations appeared to be in an advantageous position to realise plans that would strengthen their cyber defences. Elements from various prominent works discussed earlier have partially been adopted. Overall, participating organisations appeared to possess aspirations to continuously improve their sociotechnical defences that are in place. However, organisational investment for individual and skill development occurs when it is aligned with the job function being performed by the person rather than nurturing in-house talent. Our findings show:

- Positive attitude towards technologies and people can serve to build robust defences against UIT
- Subjective norms were a strong baseline but can be further reinforced through tools that allow adequate time and human centric approaches to be adopted perpetuate desirable behaviour
- Strong perceived control can be exist through the use of technologies and on individual levels however, challenges can emerge from tackling larger systems (such as resources and capacity within organisations and when people are viewed in groupings) or senior decision makers (due to time constraints or from finding effective ways of meaningful communication)
- Organisational self-positioning (based on certain considerations) is critical in the accurate assessment of defences that are in place
- A deeper understanding must be established by organisations to understand types of errors (Reason 1990) and types of tasks that originate errors (Rasmussen 1983)

Potential areas for improvement involved a revision of how inputs are organised in the assessment tool, recognising alternatives to formalised training programmes and reflecting hybrid/remote work settings post Covid-19 pandemic. In conclusion, the web-based assessment tool developed from the proposed framework allowed participants to reflect and gain new perspectives for tackling UIT at their organisations.

Limitations of this work emerge from targeted sampling (Watters and Biernacki 1989) whereby this study was not open to the wider general public. Targeted sampling was used as participating organisations were difficult to reach due to the social stigma attributed to discussions about cybersecurity practices and weaknesses. As participants were recruited through NCSC i100 partners and authors’ personal networks, participating organisations also operated in highly specialised sectors. This advanced capability for cybersecurity practices would influence the findings and limit their generalisability. Due to their collaborations with NCSC, participants might also possess more knowledge and awareness pertaining to cybersecurity practices and implementation than perhaps other organisations. When the study was advertised, interested organisations were requested to get in touch with the first author thus, organisations that took part were interested in cybersecurity and/or strategically prioritised it. This organisational interest and prioritisation is reflected in the availability and participation of senior staff. It subsequently highlights the lack of coverage of organisations that are yet to identify cybersecurity as a priority within our study. Senior staff are a suitable sample in as far as they can represent and have broad oversight of their organisations. However, it can be conceded that they might only have limited visibility of day-to-day activity and consequently, some aspects of ‘work as done’ (Hollnagel 2017). Additionally, related to most results derived from qualitative research, our findings can said to be true for the state the organisations were in at the time of the study. Aforementioned factors can limit widespread applicability of findings resulting from this study.

Whilst participants’ attitudes reflected the acceptance of human fallibility, additional research can be conducted to understand and subsequently classify the nature of errors that occur i.e. slips, lapses, mistakes and, violations (Reason 1990) that result in UIT. Future research can also investigate whether aforementioned errors emerge from particular cognitive tasks that involve skill, rule or knowledge based actions (Rasmussen 1983). Another possible opportunity for subsequent research might also examine challenges that emerge from creating and strengthening sociotechnical cyber defences at an organisational level.

## Supplementary Information

Below is the link to the electronic supplementary material.

Supplementary file1 (DOCX 121 KB)

## Data Availability

No datasets were generated or analysed during the current study.
