# Supplementary material for: Reflective interventions for cybersecurity: insights from a sociotechnical framework application and assessment
Source: Cogn Technol Work. 2025 Sep 29;28(1):113–33. doi: 10.1007/s10111-025-00833-6 (PMC13135519; doi:10.1007/s10111-025-00833-6)
Supplement: Supplementary file 1 — Supplementary file1 (DOCX 121 KB) [file 10111_2025_833_MOESM1_ESM.docx]

**Appendix A: Pre-session Questionnaire Sheet**

Q1. What are the top three priority cybersecurity areas for your organisation?

| 1. | 2. | 3. |
| --- | --- | --- |

Q2. What three words/phrases would you use to describe your organisation’s cybersecurity state right now?

| 1. | 2. | 3. |
| --- | --- | --- |

**Please select respective boxes below to indicate your preference for the following statements:**

Q3. Training and technical cyber defences (such as firewalls) are the main way to prevent Unintentional Insider Threats.

Strongly Disagree 1  2 3 4 5 6 7 Strongly Agree

Q4. Cybersecurity vulnerabilities don’t tend to change drastically over short periods of time.

Strongly Disagree 1  2 3 4 5 6 7 Strongly Agree

Q5. Unintentional Insider Threat arises as a direct consequence of users not following prescribed procedures.

Strongly Disagree 1  2 3 4 5 6 7 Strongly Agree

Q6. Cybersecurity is mainly concerned with computer-based interactions.

Strongly Disagree 1  2 3 4 5 6 7 Strongly Agree

Q7. Good cybersecurity practices and near-misses are regularly shared in the organisation.

Strongly Disagree 1  2 3 4 5 6 7 Strongly Agree

Q8. It is everyone's responsibility to be aware of cybersecurity challenges faced by the organisation.

Strongly Disagree 1  2 3 4 5 6 7 Strongly Agree

Q9. People in the organisation generally take action if they identify a cybersecurity vulnerability.

Strongly Disagree 1  2 3 4 5 6 7 Strongly Agree

Q10. It is expected of me to implement best practices to make the organisation more cyber resilient.

Strongly Disagree 1  2 3 4 5 6 7 Strongly Agree

Q11. Cybersecurity is always considered in the organisation when decisions are made about changes to procedures and resource allocation.

Strongly Disagree 1  2 3 4 5 6 7 Strongly Agree

Q12. Who in the organisation is responsible for cybersecurity?

|  |
| --- |

Q13. I am able to implement new procedures to streamline processes or enhance existing practices.

Strongly Disagree 1  2 3 4 5 6 7 Strongly Agree

Q14. I am able to start new group activities that are in the interest of the company (such as ‘Cake Fridays’ to increase morale or Equality, Diversity and Inclusion groups).

Strongly Disagree 1  2 3 4 5 6 7 Strongly Agree

Q15. I am able to take new findings from my experiences to the Board/Senior Management Team for review to inform future organisational strategies.

Strongly Disagree 1  2 3 4 5 6 7 Strongly Agree

Q16. The main way of avoiding Unintentional Insider Threats related cyberbreaches is through restricting what users can do with organisation’s IT systems.

Strongly Disagree 1  2 3 4 5 6 7 Strongly Agree

**Appendix B: Post-Session Questionnaire Sheet**

Q1. In the future what will be the top three priority cybersecurity areas for your organisation?

| 1. | 2. | 3. |
| --- | --- | --- |

Q2. What three words would you use to describe your organisation’s cybersecurity state right now?

| 1. | 2. | 3. |
| --- | --- | --- |

**Please select one box for each answer below to indicate your preference for the following statements:**

Q3. Training and technical cyber defences (such as firewalls) are the main way to prevent Unintentional Insider Threats.

Strongly Disagree 1  2 3 4 5 6 7 Strongly Agree

Q4. Cybersecurity vulnerabilities don’t tend to change drastically over short periods of time.

Strongly Disagree 1  2 3 4 5 6 7 Strongly Agree

Q5. Unintentional Insider Threat arises as a direct consequence of users not following prescribed procedures.

Strongly Disagree 1  2 3 4 5 6 7 Strongly Agree

Q6. Cybersecurity is mainly concerned with computer-based interactions.

Strongly Disagree 1  2 3 4 5 6 7 Strongly Agree

Q7. Knowledge sharing at the organisation will increase in the future whereby users share cybersecurity practices and (near-miss) experiences more frequently.

Strongly Disagree 1  2 3 4 5 6 7 Strongly Agree

Q8. There will be wide interest within the organisation around insights from the personalised report.

Strongly Disagree 1  2 3 4 5 6 7 Strongly Agree

Q9. I will explore ideas to strengthen defences that are low readiness levels in the personalised report.

Strongly Disagree 1  2 3 4 5 6 7 Strongly Agree

Q10. Board members and senior staff will support my initiatives to focus on specific parts of the defences to make the organisation more cyber resilient.

Strongly Disagree 1  2 3 4 5 6 7 Strongly Agree

Q11. Workload, procedures and resources are going to be considered more closely when creating cybersecurity practices within the organisation.

Strongly Disagree 1  2 3 4 5 6 7 Strongly Agree

Q12. In the future who in the organisation should be responsible for cybersecurity?

|  |
| --- |

Q13. It is important to evaluate existing procedures and practices to become more cyber resilient.

Strongly Disagree 1  2 3 4 5 6 7 Strongly Agree

Q14. It is likely that the organisation will form a group that meet periodically to exchange information and experiences about cybersecurity.

Strongly Disagree 1  2 3 4 5 6 7 Strongly Agree

Q15. The Board/Senior Management Team will be interested in the findings shown in the personalised report to inform future organisational strategies.

Strongly Disagree 1  2 3 4 5 6 7 Strongly Agree

Q16. The main way of avoiding Unintentional Insider Threats related cyberbreaches is through restricting what users can do with organisation’s IT systems.

Strongly Disagree 1  2 3 4 5 6 7 Strongly Agree

**Appendix C: Open-ended questions used as part of a semi-structured group interview**

Q1. Going pillar by pillar, can you share any insight(s) you gained from your personalised report?

Q2. Going pillar by pillar, can you share an insight(s) you gained from the type of factors that influence UIT?

Q3. What challenges do you foresee in implementing changes to strengthen defences that are highlighted in the personalised report?

Q4. Did you enjoy today’s experience?

Q5. What did you find interesting?

Q6. Can you share something you liked?

Q7. Can you share something you did not like?

Q8. Were there things you were expecting to see that were not covered?

Q9. Were there things in the website that surprised you?
